# Bleeding From a Ruptured, Extraluminally Growing Gastric Gastrointestinal Stromal Tumor Treated by Transcatheter Arterial Embolization: A Case Report

**DOI:** 10.7759/cureus.52394

**Published:** 2024-01-16

**Authors:** Hiroshi Okano, Atsuhiro Nakatsuka, Masaomi Ogura, Katsumi Mukai, Akira Nishimura, Kana Asakawa, Youichirou Baba, Tetsuya Murata, Seiichi Hirota

**Affiliations:** 1 Gastroenterology, Suzuka General Hospital, Suzuka, JPN; 2 Radiology, Suzuka General Hospital, Suzuka, JPN; 3 Surgery, Suzuka General Hospital, Suzuka, JPN; 4 Pathology, Suzuka General Hospital, Suzuka, JPN; 5 Surgical Pathology, Hyogo Medical University School of Medicine, Nishinomiya, JPN

**Keywords:** embolization, transcatheter arterial angiography, the platelet-derived growth factor receptor alpha (pdgfra) mutation, intra-abdominal bleeding, gastrointestinal stromal tumor

## Abstract

A 49-year-old man with abdominal pain was referred to our hospital. Abdominal computed tomography showed an extraluminal tumor near the gastric anterior wall and intra-abdominal fluid collection. A ruptured intra-abdominal tumor was suspected, and emergency abdominal angiography was performed. Hemorrhage into the abdominal cavity was seen, and transcatheter arterial embolization (TAE) was performed, which stopped the bleeding. The tumor was surgically resected, and a diagnosis of an extraluminally growing gastric gastrointestinal stromal tumor was made. TAE should be considered for rare cases of extraluminally growing tumors with intra-abdominal hemorrhage.

## Introduction

Gastrointestinal stromal tumors (GISTs) are the most common mesenchymal tumors involving the gastrointestinal (GI) tract, with an incidence of ~1.2 per 105 individuals per year [1]. Localized GISTs are curable, and surgery is the standard treatment [1]. However, some GISTs and their metastatic lesions may cause acute symptoms, such as GI or intra-abdominal bleeding, requiring urgent surgical resection, endoscopic intervention, or transcatheter arterial embolization (TAE) [2-11]. Though acute bleeding is one of the complications associated with GISTs, the most common mode of bleeding is GI bleeding, and extraluminal bleeding is rare [12-14]. In Japan, 21 cases of extraluminal bleeding from GISTs have been reported, and of the 21 cases, only two cases underwent emergency TAE for the bleeding [13]. Thus, it is assumed that most cases of extraluminal bleeding from GISTs have undergone emergency surgical resection so far. However, TAE is a less invasive procedure than surgery and is effective for other gastric malignant tumor-related GI bleeding [15,16]. This report describes a case of intra-abdominal bleeding from a ruptured, extraluminally growing gastric GIST in which TAE stopped the bleeding, and the tumor was then later resected surgically. We staged GISTs using Fletcher’s and Miettinen’s classification systems. And we determined a conversion factor of 0.34 to convert serum ALP levels measured by the Japan Society of Clinical Chemistry (JSCC) method to corresponding values measured by the International Federation of Clinical Chemistry and Laboratory Medicine (IFCC) method.

Brief summary

We experienced hemostasis achieved through TAE for an extraperitoneally ruptured gastric GIST presenting with intra-abdominal hemorrhage. This case highlights the potential of transcatheter arterial angiography and embolization for rapid diagnosis of the bleeding site and effective hemostasis in GISTs with intra-abdominal bleeding.

## Case presentation

A 49-year-old man was referred to our hospital complaining of epigastralgia, right-sided abdominal pain, and shoulder pain. His epigastralgia had started two days earlier and had continued. On admission, his pulse rate was 80 beats/minute, and his blood pressure was 159/100 mmHg. His palpebral conjunctivae were normal in color, and there was no yellowish discoloration of his bulbar conjunctiva. His abdomen was flat and soft, but he had tenderness in both the epigastric region and the right hypochondrium. On abdominal palpation, there was no clear abdominal mass lesion. He had no history of abdominal trauma or abdominal bruises.

**Figure 1:**
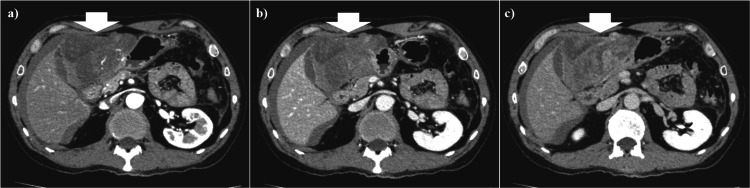
Abdominal computed tomography (CT) on admission showing an intra-abdominal peri-hepatic fluid collection with high density, which could suggest hemoperitoneum. (a) Early phase. (b) Portal phase. (c) Delayed phase. Contrast-enhanced abdominal CT shows a large heterogeneous mass with a non-uniform enhancement pattern (white arrow) at the extraluminal space of the gastric anterior wall and intra-abdominal peri-hepatic fluid collection.

Contrast-enhanced abdominal computed tomography (CT) showed a large heterogeneous tumor with a non-uniform enhancement pattern in the extraluminal space of the gastric anterior wall and intra-abdominal peri-hepatic fluid collection (Figures 1a-1c). Though a ruptured intra-abdominal mass lesion with intra-abdominal bleeding was suspected, the primary lesion of the mass could not be diagnosed. Because the patient was hemodynamically stable and the laboratory data on admission showed mild anemia and an increased C-reactive protein (CRP) level (Table 1), abdominal angiography was performed to diagnose the mass lesion and to treat it with arterial embolization if bleeding from it continued.

**Table 1:** Laboratory data on admission *Serum ALP levels measured using the IFCC method could be calculated as 0.34 times the ALP levels measured using the JSCC method. CBC, complete blood count; WBC, white blood cells; RBC, red blood cells; PT, prothrombin time; PT-INR, prothrombin time-international normalized ratio; APTT, activated partial thromboplastin time; TP, total protein; Alb, albumin; AST, aspartate aminotransferase; ALT, alanine aminotransferase; LDH, lactate dehydrogenase; ALP, alkaline phosphatase; g-GT, g-glutamyltransferase; T-Bil, total bilirubin; T-Chol, total cholesterol; BUN, blood urea nitrogen; UA, uric acid; Crea, creatinine; CRP, C-reactive protein; HBsAg, hepatitis B surface antigen; COI, cut-off index; HCVAb, anti-hepatitis C virus antibody; HIV, human immunodeficiency virus; IFCC, International Federation of Clinical Chemistry and Laboratory Medicine; JSCC, Japan Society of Clinical Chemistry.

	Reference range	On admission
CBC		
WBC (/mL)	3900-9800	7400
RBC (/mL)	427-570	387 × 10^4^
Hemoglobin (g/dL)	13.5-17.6	12.3
Hematocrit (%)	39.8-51.8	35.4
Platelets (/mL)	130-369	23.0 × 10^4^
Coagulation		
PT (%)	70-130	94
PT-INR	0.8-1.2	1.04
APTT (s)	25-45	26.7
Fibrinogen (mg/dL)	200-400	521
Chemistry		
TP (g/dL)	6.5-8.5	7.3
Alb (g/dL)	4.1-5.3	4.2
AST (IU/L)	10-35	14
ALT (IU/L)	10-35	14
LDH (IU/L) (IFCC)	124-222	112
ALP (IU/L) (IFCC)	72-113	78^*^
g-GT (IU/L)	8-60	27
T-Bil (mg/dL)	0.2-1.3	0.9
T-Chol (mg/dL)	150-219	150
BUN (mg/dL)	9.6-22.0	15.5
UA (mg/dL)	2.0-6.9	6.1
Crea (mg/dL)	0.50-1.10	0.76
CRP (mg/dL)	0.00-0.30	7.37
Viral markers		
HBsAg (COI)	0.00-0.99	0.37
HCVAb (COI)	0.0-0.9	0.0
HIV		(-)

**Figure 2:**
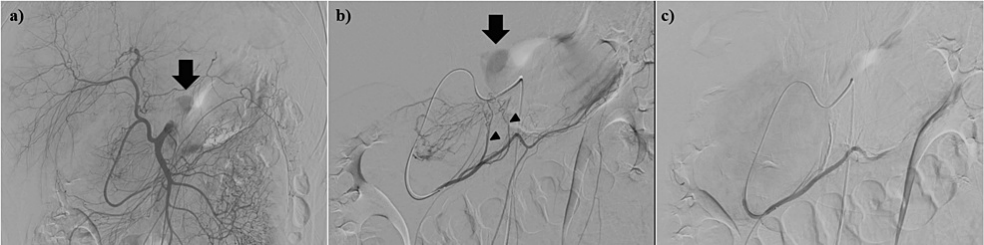
Abdominal angiography of the tumor. (a) Supra-mesenteric artery angiography. The arrow shows extravasation of contrast. (b) Selected right gastroepiploic artery angiography. The arrow shows extravasation of contrast. The arrowhead points to the epiploic branches of the right gastroepiploic artery, which supplies blood to the tumor. (c) Selective right gastroepiploic artery angiography post-embolization. Tumoral arterial extravasation is absent following embolization.

Abdominal angiography showed extravasation of contrast in the abdominal cavity (Figures 2a-2c). The blood supply of the tumor was from the epiploic branch of the right gastroepiploic artery, and the angiography showed extravasation from the epiploic branch. Based on the angiographic findings, the tumor appeared to be of gastric or greater omental origin. Because of the extravasation from the tumor-feeding artery, TAE was performed to the epiploic branch with a porous gelatin sponge (Serescue®: Astellas Pharma Inc., Tokyo, Japan). After embolization, selective right gastroepiploic artery angiography showed no extravasation from the tumor-feeding artery. Following the procedure, the patient had no abdominal symptoms, and his vital signs were stable.

**Figure 3:**
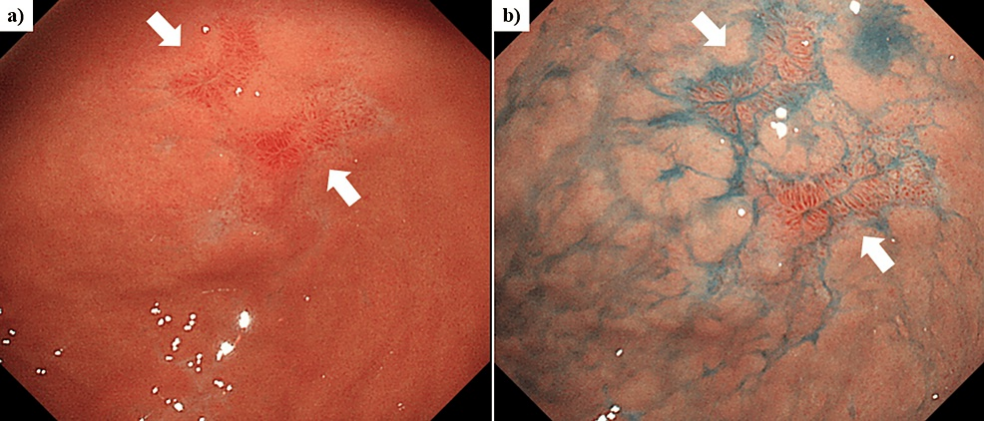
Esophagogastroduodenoscopy (EGD) showing a mild erosive lesion at the antrum (arrow). (a) Regular white light endoscopic image. (b) Chromoendoscopic image with indigo carmine staining of the gastric mucosal surface.

**Figure 4:**
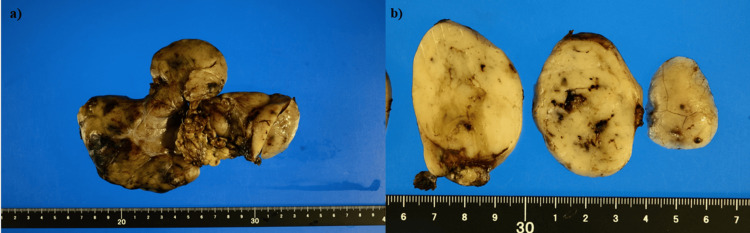
(a) Surgical intervention to remove the yellowish mass. (b) The cut surface of the tumor shows a heterogeneous yellowish area and includes the hemorrhagic or necrotic regions.

**Figure 5:**
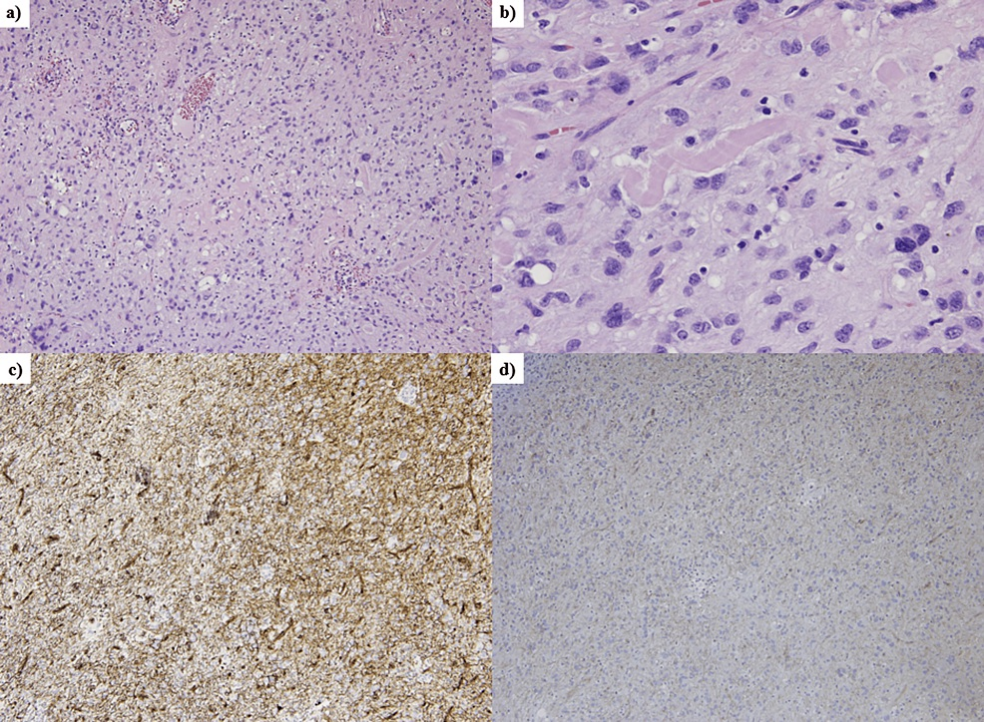
Histopathological findings. (a) Histopathological findings of the tumor stained with hematoxylin-eosin in a low-power view (×10). The tumor consists of a high density of hyperplastic tumor cells. (b) The nuclei of the tumor cells appear oval or spindle-shaped in a high-power view (×40). (c) The tumor shows positive staining for a cluster of differentiation 34 (CD34) in a low-power view (×10). (d) The tumor shows weakly positive staining for DOG1 in a low-power view (×10).

Several days later, the patient underwent esophagogastroduodenoscopy (EGD), and a mild erosion was seen surrounding the normal mucosa at the antrum (Figures 3a, 3b). The patient declined both endoscopic ultrasound (EUS) surveillance and preoperative tissue diagnosis by EUS-guided fine needle aspiration (EUS-FNA). Following TAE, the tumor underwent surgical resection (Figures 4a, 4b). The tumor was successfully dissected from the stomach, duodenum, and gallbladder, allowing for a tumorectomy. Interestingly, the hemorrhage point of the tumor could not be identified in the surgically resected specimen. The pathological findings showed myxomatous stroma with cells having epithelioid features (Figures 5a, 5b). Immunostaining of the tumor showed that the cluster of differentiation 34 (CD34) was positive and discovered GIST 1 (DOG1) was weakly positive (Figures 5c, 5d). In addition, the platelet-derived growth factor receptor alpha (PDGFRA) mutation (D842V) was detected within the tumor. Finally, the diagnosis was extraluminally growing gastric GIST with pathological findings of PDGFRA mutation in a GIST derived from gastric parietal cells and intra-abdominal hemorrhage from the GIST.

In this case, the ruptured GIST in the abdominal cavity posed a risk of disseminating tumor cells throughout the peritoneal cavity. The resected tumor, a 160 × 110 × 35 mm mass, classified as high-risk according to Fletcherʼs classification and Miettinenʼs classifications, could have also led to this outcome. However, due to the known resistance of GISTs harboring PDGFRA D842V mutations to imatinib, the patient was closely monitored without receiving adjuvant chemotherapy after surgery. Notably, even with this high-risk profile, the patient remains free of recurrent disease two years post-resection.

## Discussion

Abdominal vascular emergencies, especially intra-abdominal hemorrhage, are frequently catastrophic and highly lethal [17]. Therefore, early diagnosis and treatment of vascular emergent diseases are important. Though surgery is effective for tumor-related intra-abdominal bleeding, it is invasive and a burden for the patient. TAE offers a less-invasive alternative to surgery for tumor-related hemorrhage [15,16]. Its effectiveness stems from its ability to rapidly achieve hemostasis, even in hemodynamically unstable patients, due to its precise targeting and potent hemostatic effects. Thus, it was possible to provide prompt treatment to stop the hemorrhage in the present case by TAE. On CT imaging at admission, GIST could not be diagnosed; it could only be diagnosed after surgical resection. However, abdominal angiography was performed for the intra-abdominal bleeding from this undiagnosed tumor, and effective hemostasis was obtained. Transcatheter arterial angiography may be considered first for intra-abdominal bleeding from a cryptogenic mass lesion.

In the present case, the tumor’s origin could not be determined on contrast-enhanced abdominal CT. However, it could be assumed that the tumor was of gastric or greater omental origin because of the existence of a tumor-feeding artery from the epiploic branch of the right gastroepiploic artery. Knowing which organ is the origin of the tumor is important for surgical resection after hemostasis by embolization. Other than for GI bleeding cases, for which other useful and effective treatment procedures, such as endoscopic intervention, are indicated, TAE should be performed for bleeding from a tumor lesion as an emergency procedure.

The complications of TAE include organ ischemic injury, access site arterial trauma, contrast-induced nephropathy, and embolization of nontarget vessels [18]; therefore, TAE treatment should not be considered the only first-line option for intra-abdominal bleeding from a tumor lesion instead of surgery. However, for patients in an unstable condition, such as those with continuing hypotension or hypovolemic shock, transcatheter arterial angiography with embolization is one of the useful procedures for intra-abdominal bleeding to avoid the risks of emergency surgery and then perform elective surgery when the patient has been stabilized.

## Conclusions

Although hemorrhage is a common complication of GISTs, the intra-abdominal bleeding presented in our case differed from the typical type of GI bleeding. While endoscopic procedures may be suitable for GIST-related GI bleeding, for cases of intra-abdominal bleeding from any tumor, including GISTs, even with stable vital signs, emergency transcatheter arterial angiography and embolization should be considered as a potential approach for rapid diagnosis of the bleeding site, precise tumor localization, and effective hemostasis.
